# Health literacy profiles and empowerment strategies for older adults on an offshore island: a community-based study

**DOI:** 10.3389/fpubh.2026.1798244

**Published:** 2026-06-04

**Authors:** Ya-Ling Lin, Chun-Man Hsieh, Tsay-I Chiang, Jui-O Chen

**Affiliations:** 1 Department of Nursing, Tajen University, Pingtung, Taiwan; 2 Department of Nursing, Hungkuang University, Taichung, Taiwan

**Keywords:** community nursing, empowerment, health literacy, offshore island, older adults

## Abstract

**Background:**

Health literacy is a crucial determinant of health outcomes and reflects an individual’s ability to obtain, process, and understand essential health information for informed decision-making. Limited health literacy is associated with poor self-management, increased hospitalizations, and reduced quality of life. This study aimed to assess the level of health literacy and identify related factors among older adults residing in Liuqiu Township, a rural island community in southern Taiwan.

**Materials and methods:**

A cross-sectional study was conducted with 80 older adults aged 65 and above (74.66 ± 5.77 years). Participants were recruited from Community Care Stations through purposive sampling. Health literacy was measured using the 20-item Mandarin Multidimensional Health Literacy Questionnaire (MMHLQ). Data were analyzed using independent t-tests, one-way ANOVA, and Pearson correlation.

**Results:**

The mean total health literacy score was 2.57 ± 0.61. The highest subdomain score was found in “understanding health information” (3.06 ± 0.66), while the lowest was in “accessing health information” (2.18 ± 0.87). Participants found searching for health information on the internet particularly difficult (1.49 ± 0.89). Significant differences were observed based on education level (*F* = 19.094, *p* < 0.001, *η*^2^ = 0.33) and internet seeking behavior (*t* = 3.986, *p* < 0.001, *d* = 1.18). Notably, 90.0% of participants were on medication, while 90.0% had primary education or below. Internet search behavior was positively correlated with overall health literacy (*r* = 0.411, *p* < 0.05).

**Conclusion:**

In response to high medication use and low digital access, our team implemented a “motorcycle-based” community empowerment program. We conducted door-to-door home visits to perform personalized medication reviews and family-centered education. This proactive strategy bridges the gap between passive literacy and health empowerment, providing a foundation for nursing professionals to promote healthy aging in resource-limited rural contexts.

## 1 Introduction

Health literacy stands as a pivotal determinant of health outcomes, representing an individual’s multifaceted capacity to acquire, understand, and apply health information to promote personal health (1). It reflects a critical ability to obtain, process, and comprehend basic health information necessary for making appropriate health-related decisions, directly influencing disease prevention, health maintenance, and the effective utilization of healthcare resources (2). Beyond individual well-being, health literacy is fundamental to the functioning of public health systems. Inadequate health literacy leads to suboptimal health decisions, decreased treatment adherence, and higher hospitalization rates ultimately increasing the economic burden on healthcare systems (2–4).

Sørensen et al. (5) proposed a comprehensive integrated model, defining health literacy as the knowledge and motivation required to evaluate and apply information for healthcare, disease prevention, and health promotion. This capacity is influenced by factors such as age, education, and cognitive function; specifically, as cognitive function declines, reading comprehension and critical thinking deteriorate, further impairing an individual’s ability to manage health information (5–7). Individuals with limited health literacy often exhibit “passive compliance,” participating less in shared decision-making and showing poorer adherence to chronic disease management, such as influenza vaccination and cancer screenings (8–10). In Taiwan, recent surveys indicate that over 30% of adults face deficiencies in functional health literacy, with older adults being particularly vulnerable to polypharmacy and declined physical function due to these deficits (9, 11). With the nation projected to become a ‘super-aged society’ by 2025, the escalating complexity of disease management places unprecedented demands on health literacy (5). Furthermore, contemporary policy frameworks, such as ‘Healthy People 2030,’ have redefined health literacy as a critical social determinant of health, essential for achieving health equity and mitigating the burden on healthcare systems (12).”

To address these challenges, systematic reviews suggest that community-based interventions—ranging from traditional lectures to modern information and communication technologies (ICT) and telehealth—can significantly improve self-management skills (13, 14). For older populations, effective health promotion requires collaborative support from community hubs, such as senior activity centers, which serve as preferred and accessible learning environments (15, 16). These interventions facilitate empowerment and improve quality of life by tailoring education to the specific needs of different groups, including older adults and dialysis patients (12, 17–19).

Despite the established importance of health literacy, the unique socioeconomic and geographic landscapes of offshore island communities remain under-explored. In Liuqiu Township, 18.6% of the population is aged 65 and older, and over 86% of these seniors suffer from at least one chronic disease. Given its rural location and limited healthcare resources, older adults in Liuqiu face significant barriers to accessing health services and managing their health independently. Furthermore, the less-developed information infrastructure and limited digital proficiency among local seniors exacerbate the health information divide. To address this research gap, the present study utilized the MMHLQ to assess health literacy and its associated factors among older adults in Liuqiu Township. By identifying the needs of this “hard-to-reach” population, we aim to provide evidence-based insights for community nurses to design empowerment-based interventions and promote healthy aging in rural island contexts.

## 2 Materials and methods

### 2.1 Subjects

A cross-sectional descriptive approach was employed to explore health literacy and its associated factors among older adults in Liuqiu Township, Pingtung County, an offshore island community in southern Taiwan. Data collection took place between October and December 2023. Participants were recruited from community care centers through purposive sampling, with recruitment facilitated by community course coordinators from the Liuqiu Township Health Center during regular class sessions. A total of 80 older adults were included. Despite the modest sample size (*N* = 80), this cohort represents a significant and accessible portion of the older adult population in this geographically isolated area, providing a critical lens into a “hard-to-reach” rural community frequently overlooked in urban-centric studies.

The inclusion criteria were: (1) aged 65 years or older; (2) residing in Liuqiu Township; (3) able to communicate in Mandarin or Taiwanese; and (4) willing to provide written informed consent. Exclusion criteria included individuals with impaired consciousness, severe cognitive decline, or significant hearing/communication difficulties that hindered participation. Participants were informed of their right to withdraw at any time without penalty. Data were collected using the Mandarin Multidimensional Health Literacy Questionnaire (MMHLQ). Statistical analysis was performed using SPSS 28.0, utilizing descriptive statistics, independent *t*-tests, one-way ANOVA, and Pearson correlation analysis to identify key factors associated with health literacy. The study protocol was approved by the Ethics Committee of the Antai Hospital Institutional Review Board (TSMH IRB No. 23-083-B). Informed consent was obtained from all participants, and the study was conducted in accordance with the Declaration of Helsinki.

### 2.2 Data collection

The Mandarin Multidimensional Health Literacy Questionnaire (MMHLQ) was adopted for the health literacy survey. Developed by Wei et al. in 2017 for Taiwanese adults, this questionnaire has demonstrated strong reliability and validity and was used with authorization from the original author. The internal consistency reliability, measured by Cronbach’s *α*, reaches 0.94, while its external validity correlation coefficients range from 0.25 to 0.33, confirming it as a reliable and effective measurement tool. The MMHLQ is widely applicable not only for assessing the health literacy levels of specific populations but also for evaluating the necessity of educational interventions (9). To ensure data quality, data collection was conducted solely by two of the investigators to maintain consistency in information delivery and recording. Given that 57.5% of the participants were illiterate, data were collected through one-on-one structured interviews. The questionnaire items were read aloud in the local dialect (Taiwanese Hokkien) to ensure full comprehension, and responses were recorded by the researchers only after the participants confirmed their understanding.

### 2.3 MMHLQ questionnaire

The MMHLQ was utilized to assess multidimensional health literacy. This 20-item instrument covers five dimensions: Accessing, Understanding, Appraising, and Applying health information, and Communication and Interaction. Each item is rated on a 4-point Likert scale (1–4). According to the scoring manual, the mean scores are transformed into a 0–50 scale using the following formula:


$$\text{Standardized Score}=(\text{Mean Score}-1)\times (\frac{50}{3})$$

The resulting scores are categorized into four levels: inadequate (score≦25), limited/problematic (25 < score ≦ 33), sufficient (33 < score ≦42), and excellent (42 < score ≦ 50). The tool demonstrated excellent internal consistency in this study with a Cronbach’s alpha of 0.94.

### 2.4 Statistics

Analyses were carried out using SPSS analytic software (International Business Machines Corporation, NY, USA). Descriptive statistics, including means, standard deviations and percentages, were used to describe the participants’ characteristics and health literacy levels. Independent samples t-tests were used to compare health literacy scores between two groups (e.g., internet search behavior), while one-way ANOVA with Scheffé *post hoc* tests was employed to compare differences across three or more groups (e.g., educational levels). Pearson correlation was used to analyze the relationships between continuous variables. In addition to *p*-values, effect sizes (Cohen’s d for *t*-tests and *η*^2^ for ANOVA) and 95% confidence intervals were reported to indicate the magnitude and precision of the findings. A *p*-value < 0.05 was considered statistically significant.

### 2.5 Community empowerment program (motorcycle-based intervention)

Following the assessment, a motorcycle-based community empowerment program was proposed and implemented as a pilot strategy. This program featured a multidisciplinary team of university faculty members from the School of Pharmacy and the School of Nursing, all of whom are licensed pharmacists and registered nurses. Collaborating with local nursing staff from the offshore island township health center, this team of experts traveled by motorcycle to perform home visits. This model provided a seamless integration of academic expertise and local public health resources. Each home visit lasted approximately 20–30 min, a duration optimized to maintain the attention span of the older adults while ensuring the delivery of focused medication education.

During these visits, the team conducted clinical medication reconciliation to identify high-risk behaviors and used visual aids, such as color-coded pillboxes and customized schedules, to provide tailored education to both illiterate older adults and their family caregivers directly in their home environment. Notably, to overcome literacy barriers, we actively trained school-aged grandchildren (elementary and junior high students) within the household to act as “health assistants.” These younger family members helped bridge the information gap by reading instructions and assisting with visual aids. Preliminary qualitative observations revealed that this intergenerational approach appeared to reduce seniors’ anxiety and support their confidence in medication identification compared to clinical settings.

## 3 Results

### 3.1 Participant characteristics

A total of 80 older adults from Liuqiu Township participated in this study, with a mean age of 74.66 ± 5.77 years. Demographic analysis showed that 80.0% (*n* = 64) were female and 20.0% (*n* = 16) were male. Notably, the cohort exhibited a significant vulnerability in educational attainment: 57.5% (*n* = 46) were illiterate, and 32.5% (*n* = 26) had only completed elementary school. Combined, a staggering 90.0% (*n* = 72) of the participants had an educational level of primary school or below. This low educational background is a critical characteristic of this offshore island population. Regarding health-related behaviors, 90.0% (*n* = 72) of the participants were currently taking medications, The prevalence of chronic conditions was high among the participants; the most common conditions were hypertension (61.3%, *n* = 49), followed by diabetes (31.3%, *n* = 25), hyperlipidemia (21.3%, *n* = 17), and arthritis or osteoporosis (each at 12.5%, *n* = 10). Conversely, no cases of lung disease or cancer were reported (0.0%).while only 17.5% (*n* = 14) reported engaging in online health information seeking. The demographic characteristics of the participants are presented in Table 1.

**Table 1:** Demographic characteristics of participants (*N* = 80).

Characteristic	Category	Number (f)	Percentage (%)	Mean ± SD
Gender	Male	16	20.0	
	Female	64	80.0	
Age (years)				74.66 ± 5.77
Education level	Illiterate	46	57.5	
	Elementary school	26	32.5	
	Junior high school and above	8	10.0	
Medication use	Yes (Taking medications)	72	90.0	
	No	8	10.0	
Online health seeking	Yes	14	17.5	
	No	66	82.5	
Chronic conditions^*^	Hypertension	49	61.3	
	Diabetes	25	31.3	
	Hyperlipidemia	17	21.3	
	Arthritis	10	12.5	
	Osteoporosis	10	12.5	
	Heart disease	8	10.0	
	Others	14	17.5	

*Multiple responses allowed. Others includes gout, liver disease, kidney disease, and prostate hyperplasia.

### 3.2 Participants’ current status of health literacy

The Health Literacy Scale used in this study consists of 20 items divided into five dimensions: (1) accessing health information, (2) understanding health information, (3) appraising health information, (4) applying health information, and (5) communication and interaction. Each item was rated on a 4-point Likert scale (1 = very difficult to 4 = very easy), with higher scores indicating higher perceived health literacy. The participants’ item-level and dimensional performance are summarized in Tables 2, 3. In the dimension of accessing health information, the mean score was 2.18, indicating moderate difficulty. Participants reported the highest ease in obtaining information related to daily health care, but found it most difficult to search for necessary health information on the internet. In understanding health information, the mean score was 3.06, the highest among all five dimensions, suggesting that participants generally found it easy to comprehend and follow instructions from healthcare professionals. For appraising health information, the mean score was 2.36, reflecting moderate ability to judge the accuracy and suitability of health information; the lowest-rated item was evaluating the trustworthiness of online information. In applying health information, participants had an average score of 2.36, showing moderate ability to use health information in disease management. Finally, in communication and interaction, participants averaged 2.90, indicating that they generally found it easier to communicate with healthcare professionals than to proactively request specific tests or treatments. Overall, the total mean score of health literacy was 2.57 ± 0.61. Among the five dimensions, participants scored the highest in understanding health information and the lowest in Accessing health information (Table 3).

**Table 2:** Mean scores for the 20 health literacy items (five dimensions) (*N* = 80).

Dimension/item	Question	Mean	SD	Rank
Accessing health information	1. For me, searching for knowledge about diseases is…	2.41	1.10	2
	2. For me, obtaining information related to daily health care is…	2.48	1.08	1
	3. For me, searching for necessary health information on the internet is…	1.49	0.89	4
	4. For me, after receiving health checkup reports, further collecting related information is…	2.35	1.04	3
Understanding health information	5. For me, understanding the instructions on the medication label is…	2.69	1.10	4
	6. For me, being able to follow the instructions provided by healthcare professionals to manage diseases is…	3.28	0.57	1
	7. For me, understanding the explanations provided by healthcare professionals is…	3.20	0.60	2
	8. For me, being able to follow the instructions on the medication label to use the medication is…	3.08	0.94	3
Appraising health information	9. For me, determining whether the health information I have obtained can solve health problems is…	2.59	0.94	1
	10. For me, determining whether the health information I have obtained is suitable for my own needs is…	2.59	0.95	1
	11. For me, determining whether the health information I have obtained is consistent with other information is…	2.41	0.99	3
	12. For me, determining whether health information on the internet is trustworthy is…	1.86	0.85	4
Applying health information	13. For me, applying health information to understand changes in my condition is…	2.38	0.93	3
	14. For me, applying health information to prepare for facing a disease is…	2.39	0.92	2
	15. For me, applying health information to understand health check results is…	2.24	0.92	4
	16. For me, applying health information to choose treatment methods is…	2.43	0.96	1
Communication and interaction	17. For me, it is easy to ask the physician for the tests or treatments I want.	2.84	0.83	4
	18. For me, it is important to confirm with healthcare professionals whether I have understood the medical instructions correctly.	2.86	0.67	3
	19. For me, discussing treatment methods with the physician is…	2.91	0.75	2
	20. For me, when I have questions about explanations provided by healthcare professionals, I am able to ask questions.	2.98	0.78	1

*Each item was rated on a 4-point Likert scale (1 = very difficult to 4 = very easy).

**Table 3:** Descriptive summary of health literacy and its five dimensions (*N* = 80).

Dimension	Mean	SD	Rank
Health literacy total scale	2.57	0.61	
Accessing health information	2.18	0.87	5
Understanding health information	3.06	0.66	1
Appraising health information	2.36	0.76	3
Applying health information	2.36	0.85	3
Communication and interaction	2.90	0.59	2

*Higher scores indicate higher perceived health literacy.

### 3.3 Differences in health literacy by gender and education level

An independent samples *t*-test was conducted to examine gender differences in overall health literacy and its five dimensions. As shown in Table 4, no statistically significant gender differences were found in either the total health literacy score or any of the five subdomains (*p* > 0.05). This indicates that health literacy levels are consistent across genders in this offshore island population.

**Table 4:** Comparison of health literacy by gender and educational level (*N* = 80).

Dimension	Gender	*n*	Mean (M)	SD	*t*	*p*
Accessing health information	Male	16	2.16	0.96	−0.128	0.899
	Female	64	2.19	0.85		
Understanding health information	Male	16	3.00	0.56	−0.401	0.690
	Female	64	3.07	0.69		
Appraising health information	Male	16	2.36	0.80	−0.018	0.985
	Female	64	2.36	0.76		
Applying health information	Male	16	2.30	0.89	−0.311	0.756
	Female	64	2.37	0.85		
Communication and interaction	Male	16	3.00	0.47	0.773	0.442
	Female	64	2.87	0.62		
Overall health literacy	Male	16	2.56	0.65	−0.064	0.949
	Female	64	2.57	0.60		

Furthermore, a one-way ANOVA was used to analyze differences across educational levels (Table 5). Significant differences were found in overall health literacy (*F* = 19.094, *p* < 0.001, *η*^2^ = 0.33) and across all five subdomains (*p* < 0.05, *η*^2^ ranging from 0.08 to 0.31). *Post-hoc* Scheffé tests revealed that participants with elementary school (Level 2) or junior high and above (Level 3) education scored significantly higher than those who were illiterate (Level 1), particularly in the domains of accessing, understanding, appraising, and applying health information (all 2 > 1, 3 > 1). The 95% confidence intervals (CIs) for overall health literacy further illustrate these disparities, with the illiterate group scoring between [2.13, 2.43], compared to [2.91, 3.51] for the highest education group. While the “Communication and Interaction” domain showed a statistically significant difference in the ANOVA model (*p* = 0.042), *post-hoc* comparisons did not reach significance.

**Table 5:** Comparison of health literacy across educational levels (*N* = 80).

Dimension/education level	*n*	*M*	*SD*	*F*	*p*	Scheffé *post-hoc*	*η* ^2^	95% CI
Accessing health information				9.318	<0.001**	2 > 1, 3 > 1	0.19	
(1) Illiterate	46	1.86	0.80					[1.62, 2.10]
(2) Elementary school	26	2.53	0.68					[2.25, 2.81]
(3) Junior high & above	8	2.88	1.03					[2.02, 3.74]
Understanding health information				13.651	<0.001**	2 > 1, 3 > 1	0.26	
(1) Illiterate	46	2.78	0.63					[2.59, 2.97]
(2) Elementary school	26	3.38	0.51					[3.17, 3.59]
(3) Junior high & above	8	3.63	0.38					[3.31, 3.95]
Appraising health information				14.301	<0.001**	2 > 1, 3 > 1	0.27	
(1) Illiterate	46	2.04	0.73					[1.82, 2.26]
(2) Elementary school	26	2.68	0.57					[2.45, 2.91]
(3) Junior high & above	8	3.16	0.44					[2.79, 3.53]
Applying health information				16.959	<0.001**	2 > 1, 3 > 1	0.31	
(1) Illiterate	46	1.97	0.75					[1.74, 2.20]
(2) Elementary school	26	2.76	0.70					[2.48, 3.04]
(3) Junior high & above	8	3.25	0.48					[2.85, 3.65]
Communication & interaction				3.310	0.042*	NS	0.08	
(1) Illiterate	46	2.76	0.54					[2.60, 2.92]
(2) Elementary school	26	3.07	0.67					[2.80, 3.34]
(3) Junior high & above	8	3.16	0.48					[2.76, 3.56]
Overall health literacy total scale				19.094	<0.001**	2 > 1, 3 > 1	0.33	
(1) Illiterate	46	2.28	0.51					[2.13, 2.43]
(2) Elementary school	26	2.88	0.52					[2.67, 3.09]
(3) Junior high & above	8	3.21	0.36					[2.91, 3.51]

*p < 0.05, **p < 0.01. NS, Non-significant; *n*, sample size; *M*, Mean; SD, Standard Deviation; *η*^2^, Eta squared; CI, Confidence Interval.

### 3.4 Differences by internet health information searching

An independent samples *t*-test was conducted to compare health literacy scores between participants who searched for health information online and those who did not. As shown in Table 6, participants who utilized the internet for health information seeking (*n* = 14) had significantly higher total health literacy scores (*M* = 3.11 ± 0.54) compared to those who did not [*n* = 66, *M* = 2.46 ± 0.56; *t* = 3.986, *p* < 0.001, *d* = 1.18, 95% CI (0.33, 0.97)].

**Table 6:** Comparison of health literacy by internet health information searching (*N* = 80).

Dimension	Internet Search	*n*	*M*	*SD*	*t*	*p*	*d*	95% CI
Accessing information	Yes	14	2.86	0.82	3.414	0.001**	1.01	[0.34, 1.30]
	No	66	2.04	0.81				
Understanding information	Yes	14	3.46	0.53	2.625	0.010*	0.78	[0.12, 0.87]
	No	66	2.97	0.66				
Appraising information	Yes	14	2.93	0.49	3.244	0.002**	0.96	[0.26, 1.11]
	No	66	2.24	0.76				
Applying information	Yes	14	3.02	0.68	3.424	0.001**	1.01	[0.33, 1.26]
	No	66	2.22	0.82				
Communication	Yes	14	3.29	0.55	2.807	0.006**	0.83	[0.14, 0.82]
	No	66	2.81	0.57				
Overall health literacy	Yes	14	3.11	0.54	3.986	<0.001	1.18	[0.33, 0.97]
	No	66	2.46	0.56				

*p < 0.05, **p < 0.01. NS, Non-significant; *n*, sample size; *M*, Mean; SD, Standard Deviation; *η*^2^, Eta squared; CI, Confidence Interval.

Significant differences were consistently found across all five subdomains, with large effect sizes observed in each category (*d* ranging from 0.78 to 1.01): Accessing (*t* = 3.414, *p* = 0.001), Understanding (*t* = 2.625, *p* = 0.010), Appraising (*t* = 3.244, *p* = 0.002), Applying (*t* = 3.424, *p* = 0.001), and Communication and Interaction (*t* = 2.807, *p* = 0.006). These findings indicate that digital engagement is a critical determinant of health literacy, highlighting the vulnerability of the 82.5% of participants who remain digitally excluded.

### 3.5 Correlation analysis

Pearson correlation analysis revealed that the overall health literacy score was strongly and positively correlated with all five subdomains, including Accessing (*r* = 0.875, *p* < 0.01), understanding (*r* = 0.736, *p* < 0.01), appraising (*r* = 0.899, *p* < 0.01), applying (*r* = 0.874, *p* < 0.01), and communication and interaction (*r* = 0.620, *p* < 0.01).

As shown in Table 7, age was negatively correlated with overall health literacy (*r* = −0.296, *p* < 0.01), indicating that health literacy scores tend to decrease with advancing age among participants. Conversely, online health information seeking was significantly and positively correlated with health literacy (*r* = 0.411, *p* < 0.01), suggesting that digital engagement may serve as a facilitator for enhancing health literacy in this population.

**Table 7:** Correlation matrix of overall health literacy, its subdomains, and related variables (*N* = 80).

Variable	Accessing health information	Understanding health information	Appraising health information	Applying health information	Communication and interaction	Age	Internet search
Overall health literacy	0.875**	0.736**	0.899**	0.874**	0.620**	−0.296**	0.411**

**p < 0.01. (Pearson’s correlation coefficients).

## 4 Discussion

The primary aim of this study was to evaluate the multidimensional health literacy of older adults in Liuqiu Township. Our findings revealed a distinct pattern: participants demonstrated the highest competence in understanding health information (3.06 ± 0.66) but the lowest in accessing it (2.18 ± 0.87). This suggests a state of “passive health literacy” among rural island-dwelling seniors, who are capable of comprehending professional guidance once provided but face significant barriers to independent information seeking. These results are consistent with previous research identifying digital literacy and limited access to technology as barriers to health information acquisition among rural older populations (8, 9). Our findings specifically align with the core domains of Sørensen’s Integrated Model (2012). The MMHLQ dimensions of ‘Information Access’ and ‘Understanding’ used in this study directly correspond to the ‘Access’ and ‘Understand’ stages of the Sørensen model. Furthermore, the low scores observed in health information appraisal reflect challenges in the ‘Appraise’ domain, highlighting how the unique ecological barriers of the offshore island—such as geographic and digital isolation—impede the critical evaluation of health information among older adults. A recent study on the digital factors influencing of health confirms that geographic isolation and digital exclusion are universal barriers in remote settings, mirroring challenges observed in rural Australia and Japan (20, 21). Notably, the high prevalence of medication use (90.0%, *n* = 72) among participants, coupled with their low literacy (90.0% primary school or below) and limited online health information seeking behavior (17.5%, *n* = 14), underscores an urgent need for direct intervention.

In response to these critical vulnerabilities, our research team implemented a comprehensive community outreach program. Recognizing the geographical and literacy barriers of Liuqiu Township, we conducted door-to-door home visits via motorcycles to perform personalized medication reviews. During these intensive visits, we systematically inspected each participant’s medication regime and provided tailored health education to both the seniors and their family caregivers. By engaging the family unit in their own homes, we created a localized supportive environment to ensure medication safety, directly addressing the limitations identified in their “accessing” and “appraising” health literacy domains. This proactive, home-based approach aligns with international evidence suggesting that customized education delivered in the home environment can significantly mitigate the impact of low health literacy on health outcomes in aging populations (22). This intervention leverages the concept of “distributed health literacy,” where individuals with limited personal skills navigate complex health tasks by drawing on the resources of their social networks (23). In this context, family members act as “literacy mediators” who facilitate communication and assist in navigating the healthcare system (17, 18), effectively compensating for the older adults’ individual literacy barriers.

This observed discrepancy reflects a “passive compliance” pattern often seen in traditional Asian rural cultures. In these settings, older adults frequently rely on healthcare authorities but lack the proactive skills to independently navigate modern, digitalized health information systems. Consequently, this leads to a state of “digital exclusion,” where limited information-seeking behavior becomes a primary barrier to health empowerment (11, 17). This digital divide is a global phenomenon; research in Saudi Arabia also identifies digital health engagement as a critical factor associated with of health outcomes, regardless of the cultural context (24). Instead of traditional training, we suggest a “family-assisted digital navigation” approach. Our intervention successfully demonstrated the potential of involving school-aged grandchildren as digital and literacy mediators. Future programs could leverage this intergenerational dynamic by training these youth to use tablets for accessing simple, voice-activated search tools or video-based health education for their grandparents. This not only empowers the younger generation with health knowledge but also provides a culturally sensitive way to bridge the digital divide for illiterate seniors. Gender differences were not significant across the domains, while education level showed a strong positive association with health literacy (*F* = 19.094, *p* < 0.001). Participants with higher educational attainment achieved better scores, reinforcing prior evidence that education is a critical associated factor. Likewise, internet health-seeking behavior emerged as a crucial enabling factor (*t* = 3.986, *p* < 0.001), echoing findings that digital engagement supports self-directed learning (5, 6, 25). While digital literacy training is proposed as a potential strategy, traditional tablet-based education may be challenging for older adults with limited literacy. Instead, we suggest a “family-assisted digital navigation” approach. For instance, using tablets as a platform for simple, voice-activated search tools or short video-based health education modules can bridge the literacy gap. This allows nursing professionals to transition from being the sole providers of information to facilitators of a digital-supportive environment, empowering seniors through visual and auditory aids rather than complex text-based interfaces.

Moreover, these findings provide critical insights for both clinical and community nursing. In clinical settings, such as hospital outpatient departments or clinics, healthcare providers should move beyond providing written pamphlets. Instead, they should utilize the ‘Teach-Back’ method in the local dialect to verify that seniors with ‘passive health literacy’ truly comprehend medication instructions before leaving the consultation room. In community settings, while door-to-door visits are a standard practice in local public health centers, our study suggests optimizing these encounters by transitioning to family-centered ‘health navigation.’ By engaging family members as literacy mediators, nursing professionals can bridge the information divide and ensure safer home-based medication management (11, 17, 18, 20). The development of age-appropriate, culturally sensitive materials is essential to bridge the health information divide in offshore island communities (15, 16).

Several limitations should be acknowledged. The relatively small sample size (*N* = 80) and its geographic restriction limit generalizability. Notably, 80.0% of the participants were female, creating a significant gender imbalance. However, this distribution aligns with the documented social reality in Pingtung County; according to the latest official statistics from the Pingtung County Government (2025), older women demonstrate significantly more proactive and consistent participation in community care center services compared to their male counterparts. While the smaller male sample size (*n* = 16) may reduce the statistical power to detect subtle gender differences, these findings offer a preliminary yet realistic snapshot of the population currently reached by community-based health initiatives in such offshore island settings. Research in such resource-limited and geographically isolated environments is inherently challenging. Despite the modest sample size, this cohort represents a significant and accessible portion of an offshore community frequently overlooked in urban-centric studies. Future studies are encouraged to employ larger samples to explore potential causal pathways between health literacy and health outcomes (13, 14).

Despite these limitations, this study contributes valuable evidence to the understanding of health literacy in rural island communities. The findings emphasize that education and digital inclusion are fundamental to promoting self-care, providing a foundation for nursing professionals to design interventions that foster empowerment and healthy aging in resource-limited settings (14, 19).

## 5 Conclusion

This study demonstrates that older adults in this offshore island community exhibit a distinct “passive health literacy” profile, characterized by adequate comprehension of health instructions but significant barriers to independent information acquisition. Education level (*F* = 19.094) and digital engagement (*t* = 3.986) emerged as pivotal factors associated with health autonomy, emphasizing the urgent need to bridge the digital divide in rural aging populations. Critically, the high prevalence of medication use (90.0%) among these low-literacy seniors underscores that traditional health education is insufficient.

These findings necessitate a strategic shift: community-based care must evolve toward a proactive, family-centered navigation model. Proposed strategies such as our “motorcycle-based” home visit initiative suggest that direct, individualized medication reviews and family-targeted education can effectively bridge the health information gap. By integrating such mobile, localized support into routine services, we can mitigate the risks of “passive compliance” and enhance seniors’ self-management capabilities. Ultimately, fostering collaborative ecosystems among healthcare providers and policymakers is essential to ensure medication safety and sustainable healthy aging in resource-limited rural contexts.

## Data Availability

The data supporting the findings of this study are available within the article and its supplementary materials, and any further inquiries can be directed to the corresponding author.
